# Protein-Based Packaging Films in Food: Developments, Applications, and Challenges

**DOI:** 10.3390/gels10070418

**Published:** 2024-06-25

**Authors:** Rui Zhang, Rongxu Liu, Jianchun Han, Lili Ren, Longwei Jiang

**Affiliations:** 1 College of Food Science, Northeast Agricultural University, Harbin 150030, China; 13293953968@163.com; 2 Heilongjiang Institute of Green Food Science, Harbin 150028, China; rongxuliu@163.com; 3 Key Laboratory of Bionic Engineering (Ministry of Education), College of Biological and Agricultural Engineering, Jilin University, Changchun 130022, China; liliren@jlu.edu.cn; 4 College of Tea & Food Science and Technology, Anhui Agricultural University, Key Laboratory of Jianghuai, Agricultural Product Fine Processing and Resource Utilization, Ministry of Agriculture and Rural Affairs, Anhui Engineering Research Center for High Value Utilization of Characteristic Agricultural Products, Hefei 230036, China

**Keywords:** active packaging, protein-based film, gel film, performance characteristics, applied research

## Abstract

With the emphasis placed by society on environmental resources, current petroleum-based packaging in the food industry can no longer meet people’s needs. However, new active packaging technologies have emerged, such as proteins, polysaccharides, and lipids, in which proteins are widely used for their outstanding gel film-forming properties. Most of the current literature focuses on research applications of single protein-based films. In this paper, we review the novel protein-based packaging technologies that have been used in recent years to categorize different proteins, including plant proteins (soybean protein isolate, zein, gluten protein) and animal proteins (whey protein isolate, casein, collagen, gelatin). The advances that have recently been made in protein-based active packaging technology can be understood by describing protein sources, gel properties, molding principles, and applied research. This paper presents the current problems and prospects of active packaging technology, provides new ideas for the development of new types of packaging and the expansion of gel applications in the future, and promotes the development and innovation of environmentally friendly food packaging.

## 1. Introduction

Product packaging is important in the food industry to extend the shelf life of perishable food such as vegetables, fruits, and meat, as well as to ensure their nutritional value and safety. Product packaging is particularly important. At present, packaging materials are still dominated by synthetic plastic films made from petroleum. Polymeric materials consisting of polyethylene, polypropylene, and polyethylene terephthalate are widely used for food packaging. These films have the advantages of low cost, good gas, and liquid barrier properties, and high durability. However, since petroleum is a non-renewable resource, long-term use exacerbates the consumption of resources, the carbon–carbon bonds in the petroleum base make this packaging non-degradable, and overuse cannot be mitigated in a short period of time, causing a serious burden on the natural environment. It is estimated that approximately 8 MMT of plastic packaging is discharged into the ocean each year because it cannot be disposed [1]. As it is discarded anywhere and is primarily white in color, it is also known as “white pollution”. At present, the main methods to deal with waste packaging are still incineration and landfill. However the former produces a large amount of carbon dioxide and other harmful substances, exacerbating environmental pressure [2], and the latter not only takes up considerable land resources but also causes certain harmful components to remain in soil for a long time, which is not conducive to its subsequent utilization. Therefore, demand has grown for finding raw materials that are natural, biodegradable, and renewable for use in produce packaging. With the intensive research on biodegradable active packaging technology, new types of food packaging based on proteins are now emerging. In particular, edible gel films made from animal and plant proteins are favored for their processability, versatility, high nutritional value, and better control of gas and aroma exchange properties [3]. The variety of amino acids contained in proteins themselves, as well as their complex spatial structure, improve the mechanical properties of protein-based films compared to novel packaging from other sources [4].

**Figure 1:**
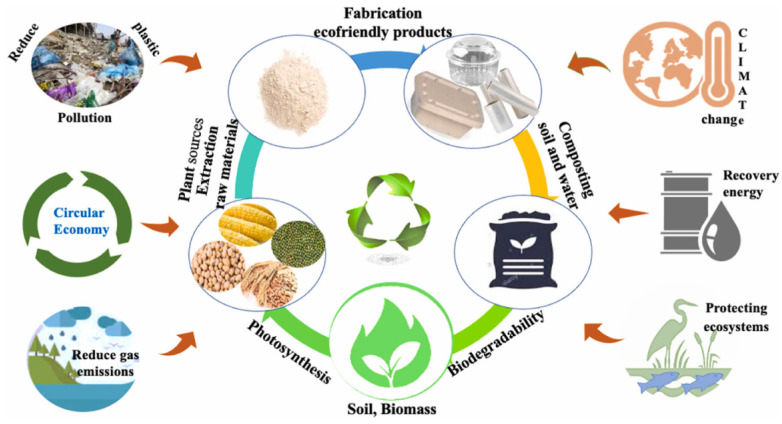
The cycle and importance of protein-based packaging [4].

**Figure 2:**
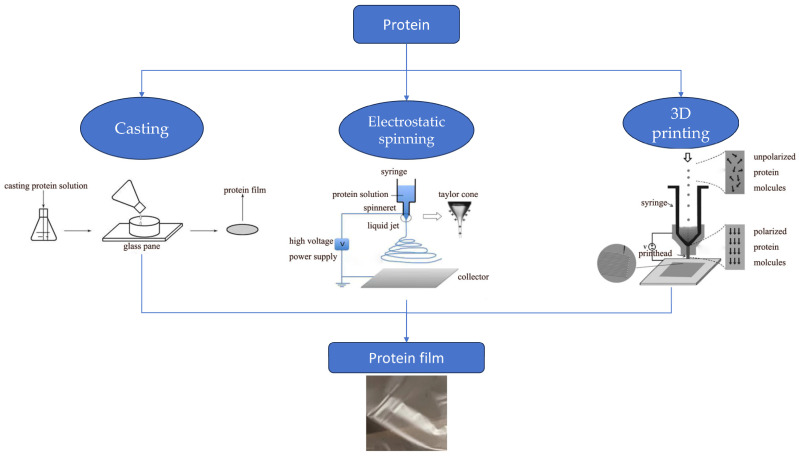
Different ways to prepare protein-based films.

As shown in Figure 1, protein films avoid or minimize damage caused by microorganisms and undesirable environments by covering the surface of food. Some of these films are directly edible and easily degrade even when buried in the soil, reducing environmental pollution. Currently, protein-based materials are divided into two main categories: plant and animal proteins. The former are mainly derived from various grains but can also be derived from oilseed products; examples include soybean protein isolate, zein, gluten protein, sunflower protein, and pea protein isolate [4]. The latter are derived from meat, eggs, and milk from livestock and fish such as whey protein, casein, collagen, and gelatin [5]. Protein gelation refers to the modification of protein molecules through the adoption of technical means of aggregation to form an orderly network structure. The gelation characteristics of the protein-based films are endowed with strong mechanical properties and excellent gas barrier function. However, at the same time, since most of the proteins are hydrophilic, the gel packaging film has poor water repellency. To overcome this, as shown in Figure 2, techniques such as casting, electrostatic spinning, and 3D printing are commonly used to remodel the protein spatial structure by incorporating nanoparticles, plasticizers, bioactives, and biopolymers into the proteins, modulating the film properties by improved protein molecular characterization to enhance the utilization of protein-based films [6].

In recent years, a variety of protein-based active packaging based on different raw materials have been developed and produced, and a variety of new packaging technologies have emerged, such as 3D printing and nanogel technology. Three-dimensional printing is also known as additive manufacturing technology. First introduced in the 1980s [7], it can streamline the supply chain and personalize products for consumers, so 3D food printing is advancing by leaps and bounds with new packaging technologies [8]. Nanogels are composed of nanostructures of hydrophilic molecules that are internally cross-linked through electrostatic interactions, hydrogen bonding, and hydrophobic bonding, and they have the common properties of gels and the small particle size and interfacial effects of nanomaterials [9]. Nanogel technology allows proteins to be formed as a liquid into new types of food packaging film with unique features [10]. Electrostatic spinning is a technique for producing nanofibers by stretching natural or synthetic polymers in the presence of an electric field. As nanoscale materials in the field of food packaging industry have great antimicrobial capacity compared with traditional materials, and electrostatic spinning technology is simple to operate and can be used continuously, it is widely used in new protein-based active packaging [11].

**Figure 3:**
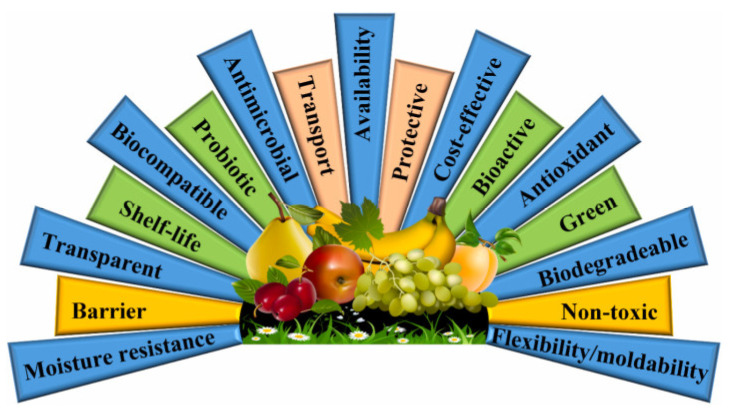
Characteristic features of protein-based food packaging materials [12].

There have been a number of articles describing protein-based films in recent years, but mostly as a small section on a new type of packaging or describing plant or animal proteins alone. Unlike other articles, this paper focuses on protein-based films in novel packaging, investigating both animal and plant protein films. As shown in Figure 3, protein-based films have some unique characteristic features. Through the study of films, it is found that their principle is based on the utilization of protein gelation and film production is carried out by means of processing modification. This can not only reduce environmental pollution to a certain extent but also help to save resources and provide ideas and a theoretical basis for subsequent research of new protein films and gel applications.

## 2. Protein Classification and Applications

### 2.1. Recycling Protein from Waste to Make Films

**Figure 4:**
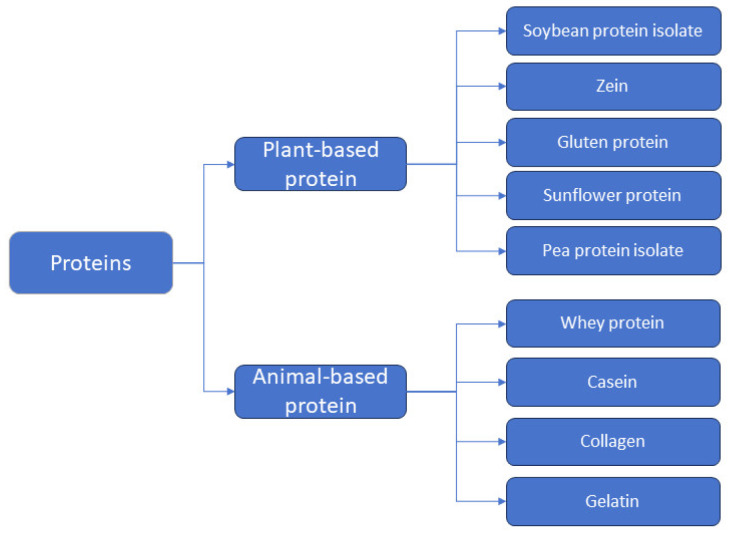
Protein types in protein-based active packaging.

**Figure 5:**
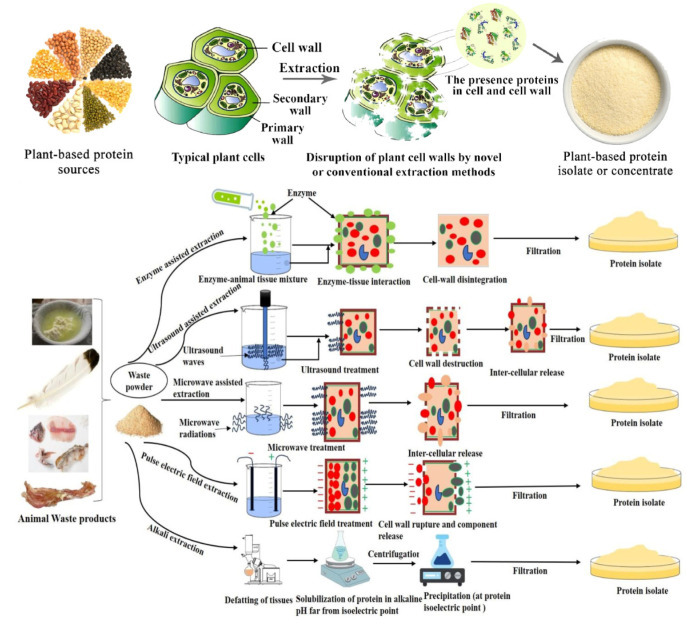
Protein recovery processes from plants and animals [3].

The emergence of protein-based films aims to reduce the impact of petroleum-based films on the environment. As shown in Figure 4, we can extract proteins from a variety of plants and animals for film preparation. At the same time, in order to better protect the environment and conserve resources, people have also placed some focus on waste containing protein and have sought to reuse this protein to avoid wasting resources [3]. The sources of waste in the food industry are the beverage industry, the dairy and ice cream industry, the fruit and vegetable industry, cereals and starch products, meat products, vegetable and animal fats, and oils and fish products. If discharged directly, wastewater from plant processing such as corn starch processing and potato processing, which often contain a large number of proteins, will lead to the eutrophication of the water body and pollution of the environment and people through the use of film separation, flocculation, and other means of protein recovery such as reprocessing and utilization. In the brewing industry, beer waste grains contain a high amount of underutilized high-quality protein. The proteins are recovered through alkali treatment and mixed with chitosan to make a composite film with antimicrobial and antioxidant activity, suitable for food packaging. Animal residues such as bones and fur are discarded in large quantities, and their decay produces a bad odor, not only polluting people’s living environment but also causing resource waste. As shown in Figure 5, by collecting the residues and extracting and recycling the proteins, they can also be used in the production of food packaging. A large portion of the world’s population suffers from malnutrition due to food shortages and the inability to consume enough protein, vitamins, and other nutrients. Recovering proteins from waste by-products to prepare packaging films is a great food-saving method, not only for a cleaner, zero-waste environment and an ample supply of proteins, but also as a mainstream trend in future film manufacturing.

### 2.2. Plant-Based Protein

#### 2.2.1. Soybean Protein Isolate (SPI)

**Figure 6:**
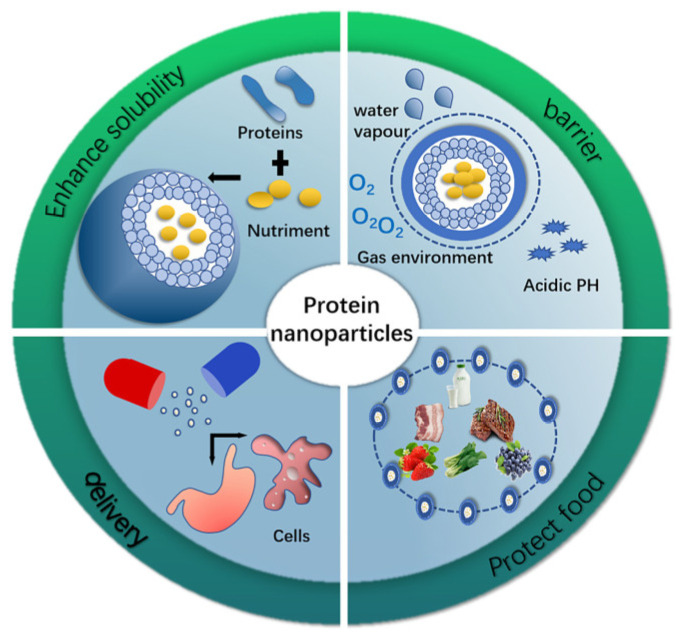
Application of protein nanoparticles [17].

SPI is a protein product made from soybeans after peeling and degreasing to remove non-protein components. It is a high-quality protein resource that is not only inexpensive but also nutritious and rich in essential amino acids required by the human body, containing >90% of globulin, predominantly β-conglycinin (7S) and glycine (11S) [13]. The SPI molecule contains a large number of hydrogen bonds, hydrophobic bonds, ionic bonds, and disulfide bonds; it exhibits better film-forming properties and degradability; and it has better application prospects in terms of regeneration in the food packaging industry [14]. However, at the same time, due to the presence of more hydrophobic amino acids in the soybean isolate protein, the protein becomes a hydrophilic layer wrapped around the hydrophobic layer of a spherical structure, causing the poor waterproof performance of the film. Moreover, the natural properties of SPI cause poor mechanical properties of soy protein-based films, which severely limit their applications. To overcome this, the spatial structure between protein molecules can be altered by means of ultrasound, heating, microwaves, etc., or as shown in Figure 6, the usability of protein gel film can be improved by combining proteins with nanoparticles, for example. The combination of cellulose nanocrystals (CNCs) and *Cedrus deodara* pine needle extract in SPI resulted in an increase in the tensile strength of the soy protein film by about 40% and a reduction in the elongation at break of the film by more than 50% [15]. Grape seed extract (GS) and green tea extract (GT) were combined with SPI to prepare protein films using 3D printing technology, which resulted in enhanced film properties with reduced clarity, increased tensile strength, and a 61% reduction in water vapor permeability [16].

Combining SPI with sodium alginate enhances the mechanical strength and environmental resistance of the film through covalent interactions [18]. Adding carvacrol and glycerin to SPI can act as a plasticizer and improve the ductility of the gel packaging film [19]. Microorganisms such as *Pseudomonas fluorescens* and *Aeromonas hydrophila* can have a significant impact on product properties during the storage and transportation of foods such as meat, which are highly susceptible to spoilage. In order to avoid food spoilage caused by microorganisms, natural compounds are often added to protein-based films to improve their bacteriostatic properties. Combining SPI, montmorillonite, and clove essential oil inhibits the growth of *Pseudomonas* strains and extends the shelf life of food products [20].

#### 2.2.2. Zein

Zein is the main by-product protein of corn processing, accounting for 70–85%, and it can be used for a variety of purposes, including food packaging, cosmetic processing, and drug transportation [21]. Because zein is itself a hydrophobic protein, zein-based films have a natural advantage over other, new films in terms of water resistance. In addition, it has been shown that zein-based gel films can be used as controlled release matrices for the delivery of active substances such as Streptomyces lactis peptides, lysozyme, and thymol [22]. The plasticizers sorbitol, glycerol, and PEG-400 were mixed with zein to make a film, whose mechanical properties and barrier capacity were enhanced as the plasticizer concentration increased [23]. A protein-based film was prepared by mixing chitosan, zein, and alpha-tocopherol, which can provide antioxidant and browning delaying effects [24]. Since proteins are susceptible to thermal denaturation, DBD cold plasma technology was applied to the production of chitosan and corn protein films, the thermal degradation temperature of the films was increased from 264 to 323.57 °C, and the thermal stability of the protein films was improved [25]. The addition of hazelnut skin powder extract and α-tocopherol as oxidants to zein enhanced the antioxidant and bacteriostatic properties of films by reducing the free acidity and peroxide value [26]. The application of 10% clove essential oil (CEO) to a 30% concentration of corn alkyd protein film by the electrostatic spinning technique significantly improved the bacteriostatic properties of the cheese [27]. It was report that a single zein-based gel packaging film was brittle and fragile, so cellulose acetate, garlic essential oil, and β-cyclodextrin were added to this film made to improve its flexibility, meeting the needs of food packaging [28]. Catechin (CA) is a natural polyphenol with antioxidant properties. The preparation of CA and β-cyclodextrin-containing nanoparticles (CINPs) of maize alkyd protein films not only makes the film surface smoother, but also increases its tensile strength from 2.28 to 12.49 MPa and increases the elongation at break from 1.52 to 4.5%. With the increase in NP dosage, the antioxidant activity of Zein/CINPs-100 increased by about 42% compared with Zein/CINPs-25, and the antioxidant property and mechanical strength of the film were enhanced, which effectively prolonged the shelf life of the foodstuffs and was more suitable for food packaging [29].

#### 2.2.3. Gluten Protein

Gluten protein is mainly derived from grains such as wheat, barley, and rye. Classified by solubility into gliadin and glutenin, the former is a soluble protein while the latter is insoluble [30]. The former has a low molecular weight ranging from 28 to 55 kDa, while the latter has a high molecular weight ranging from 30 to 140 kDa [31]. Composite gluten- protein-based gel films are processed by techniques such as drying and heat treatment as a means of enhancing film properties. The viscoelasticity of gluten protein films is mainly determined by the gliadin in gluten proteins [32]. It has been found that gluten protein films are excellent at blocking gases, meaning that these films can protect food from oxygen, moisture, and other undesirable environmental effects, and can effectively extend the freshness of food [33]. Combining sodium sulfite with gluten protein enhances the strength of the film, and the addition of calcium chloride promotes cross-linking within the film to improve its stretchability [34]. The antioxidant properties and low solubility of these films can be achieved by adding chlorophyll and polypyrrole to them [35]. The gel films prepared by mixing carvacrol and cinnamaldehyde with gluten proteins exhibit greatly improved antimicrobial properties. The chemical cross-linking of a mixture of keratin and gluten proteins to prepare gel films through the use of 3D printing can improve the elasticity, tensile strength, and mechanical properties of the film and enable it to have a wider range of applications [36].

#### 2.2.4. Sunflower Protein

Sunflower, as a major oilseed plant, is often used for oil production, and the sunflower oil cake after oil extraction is discarded as processing waste, but it is also rich in protein [37]. By recovering sunflower proteins from sunflower oil cake, the preparation of bioactive packaging based on these proteins could satisfy people’s desire to save resources and protect the environment. The alkaline treatment of sunflower seed cake allows the extraction of 11S globulin and 2S albumin, which are the main components of sunflower proteins. Sunflower protein-based films have poor mechanical properties, so the addition of plasticizers was often required to improve film properties. It has been shown that the best film elasticity was prepared by combining glycerol with sunflower protein, but there was no significant increase in film strength. By contrast, combining 1,3-propanediol with the protein resulted in the best film strength but the elasticity was not sufficient for the packaging needs [38]. Therefore, a mixture of two or more plasticizers was often added to the sunflower protein-based film to improve the properties of the film through interaction [39]. Since sunflower protein contains phenolic compounds such as chlorogenic and caffeic acid, polyphenols are tightly bound to proteins and cannot be completely removed during the processing of protein films [40]; thus, the sunflower protein-based film tends to be opaque and green, while the polyphenols impart strong antioxidant properties to the film [41]. However, the antimicrobial properties of the sunflower protein-based film are not strong enough to avoid microbial interference during food transportation and storage. To overcome this, a gel film can be made by adding clove essential oil to sunflower protein, which further enhances the antioxidant and antimicrobial properties of the film and does not cause any degradation of its other properties which can sufficiently extend the shelf life of food products [42].

#### 2.2.5. Pea Protein Isolate (PPI)

As the second largest legume in the world after soybeans, peas are a rich source of protein [43], typically containing 20 to 25% the protein extracted from peas is called pea protein isolate (PPI). PPI can be extracted by dry and wet fractionation, salt extraction, and mild fractionation. It is often considered a low-risk allergenic protein [44], and has a well-balanced amino acid composition, good dispersion, stability, and good gel properties [45]. Because of its sustainability and affordability, pea protein has also been used in the preparation of protein-based gel packaging films [46]. PPI exhibits good mechanical properties and UV-blocking ability, but its poor flexibility and water resistance limit its use as a pure compound in film processes. Various techniques such as high-pressure homogenization (HPH) and electrostatic spinning have been used to modify the structure of PPI in order to improve the properties of the films [47]. The blending of acetylated tapioca starch (AS) and PPI to prepare films by blown film extrusion technology improves the physical, thermal, and barrier properties of the film as well as the stability of oil packaging [48]. The gallic acid PPI-based nanofiber films prepared by electrostatic spinning provide excellent protection for heat-sensitive foods and greatly extend the shelf life of foods [49]. The blending of micro- and nanoemulsion-doped oregano essential oil (OEO) with PPI for film production significantly improves the water vapor barrier, mechanical properties, and bacteriostatic properties of the film [50]. The film prepared by mixing glycerin with PPI is smoother in appearance and touch, has lower light transmittance, performs well in the packaging and storage of photosensitive foods, and has a wider range of applications, while its mechanical properties can be comparable to and be used as a replacement for whey protein-based films, due to its lower cost, as it is a plant-based protein [51].

### 2.3. Animal-Based Protein

#### 2.3.1. Whey Protein (WP)

WP, a by-product of cheese production, is safe, non-toxic, and rich in β-lactoglobulin, α-lactalbumin, immunoglobulins, and lactoferrin [52]. This protein is advantageous since it is rich in nutrients, containing a variety of essential amino acids as well as calcium, potassium, magnesium, iron, and other trace elements which have important roles in human growth and development, and it has high yield and degradability. Therefore, it has attracted much attention in the food industry. WP has excellent properties such as high thermal stability, non-toxicity, and the ability to form strong internal cross-links and micelles making it an excellent choice for new active packaging technologies [53]. WP comes in the form of gels, which are denatured and aggregated by heat to form a three-dimensional network structure for wrapping other substances [54]. Films made from WP exhibit good gas barrier properties, and their antimicrobial properties and mechanical strength can be improved by incorporating chitosan nanofibers (CSNFs) into WPs. ZnO nanofilms were prepared using WP as a substrate, and it was found through testing to have significant bacteriostatic properties against *Staphylococcus epidermidis*, demonstrating the feasibility of WP-ZnO films in food packaging [55]. The gel prepared by combining konjac flour, sodium bicarbonate, and rennet enzyme with 20% whey protein showed the best 3D printing results, with the sample being highly supportive and smoothly extruded, with a homogeneous and dense internal structure as well as enhanced hardness and elasticity [56].

#### 2.3.2. Casein

Casein accounts for about 80% of the total protein content of milk [57], and it is composed of 38% αs1 casein, 36% β-casein, 13% κ-casein, 10% αs2 casein, and 3% γ-casein [58]. Casein has an isoelectric point of about 4.6, exists in the form of micelles, and is highly processable due to the low content of cysteine resulting in fewer disulfide bonds in casein, which can form arbitrary exposed structures. Casein is non-toxic, chemically resistant, and degradable. Combining these advantages, it is commonly used as a substrate for protein-based films [59]. Because it is more insoluble compared to other proteins, it is often involved in the preparation of protein-based films in the form of caseinates. Casein-based films prepared by mixing glycerol, citric acid, pectin, and calcium caseinate showed excellent performance in terms of film strength and elasticity and a reduced sensitivity to the surrounding environment, which made it easier to store food products and prolong their shelf life; the degradation of the film in the soil took only 16 days [60]. The use of films made by combining chitosan and sodium caseinate for the packaging of foodstuffs has greatly improved the antimicrobial properties of foodstuffs [61]. Meanwhile, sodium caseinate can be used not only for the preparation of both solution-and emulsion-type films in caseinate emulsion by adding surfactants or biopolymers to improve the stability of the emulsion. During processing, which is susceptible to processing conditions and environmental influences, it is usually necessary to add plasticizers to improve mechanical properties [62]. The addition of low concentrations of gallic acid to the sodium caseinate and guar gum composite film resulted in a 21% decrease in the water vapor permeability of the film and an increase in the water solubility of the film from 58% to 63%, thus making the film more biodegradable and causing a significant increase in the opacity, with a high antioxidant potential (~80% DPPH inhibition) [63]. Gel films were prepared by combining modified porous starch with casein and adding k-carrageenan as a gelling agent, which resulted in an increase in tensile strength (TS, from 42.37 to 94.05 MPa) and a decrease in elongation at break (EAB, from 13.43 to 8.48%) of the films. The moisture content (MC) of the film was reduced from 18.63 to 15.67% and its water solubility (WS) was reduced from 36.02 to 33.79% [9].

#### 2.3.3. Collagen

Collagen is an important functional protein that is not only versatile but also abundant [64]. It is obtained from both mammals and marine animals. Marine collagen has a lower molecular weight and denaturation temperature compared to mammalian collagen, and cross-linking is typically required for the former [65]. The structure of collagen consists of three cross-linked alpha chains, a fibrous protein with a typical triple-helical structure [66]. Collagen is rich in methionine, hydroxyproline/proline, and glycine [67]. Its stability is determined by the number of hydrogen bonds in its proline and hydroxyproline. Collagen films synthesized by the extrusion process usually have more applications in food packaging [68]. These films are also more stretchable than those with other matrices due to their unique triple-helix structure [69]. A blend of collagen, gallic acid, chitosan, and ε-polylysine (ε-PL) was used to produce the film, which significantly improved the antimicrobial, antioxidant, and tensile strength of the collagen-based film. Wrapping the film around pork for freshness doubled the shelf life, while embedding the used film in soil was found to degrade it in as little as 20 days [70]. Films prepared by blending antimicrobial tea polyphenols with collagen significantly improved the mechanical and antimicrobial properties of the films.

#### 2.3.4. Gelatin

Gelatin is an animal-derived protein usually available from by-products of meat product processing, such as collagen-rich animal bones, skin, tendons, and hooves. When heated under strong acidic and alkaline conditions, collagen is converted to gelatin. The difference between gelatin and other hydrophilic colloids is that it can form thermo-reversible gels at lower concentrations depending on the temperature, which determines whether the gelatin solution is in a liquid sol or solid gel state, and the two states exhibits reversibility between them [71]. In the food industry, gelatin is commonly used to prepare confectionery as a gel at low ambient temperatures, as well as in the wine and beer brewing industries [72]. Gelatin-based films can be categorized by temperature into cold-resistant films suitable for frozen storage and hot cast films. Gelatin-based films have translucency, better tensile elasticity, and antimicrobial and antioxidant properties. Because of its good performance, gelatin has also become an indispensable substrate for new packaging materials [73]. However, at the same time, protein-based films made of gelatin have poor barrier properties, especially for water vapor transport; this can be overcome by combining gelatin with other biopolymers such as agar [74] chitosan [75], and starch [76]. Currently, other materials are added to enhance the properties of the film; for example, gelatin is combined with TiO_2_ to prepare a film with excellent antimicrobial activity and UV resistance [77]. The tensile strength of the film, prepared by adding catechin, chlorogenic acid, and anthocyanin B2 to gelatin, was increased from 33.7 to 40.9 MPa; the water permeability was reduced by 40%; the opacity was improved from 0.71 to 2.14 mm^−1^; shading was enhanced; and the DPPH radical scavenging activity was increased from 33.42 to 84.40%. Films made from 4 or 6 wt% extracts can significantly extend the shelf life of pork [78]. The application of chitin nanowhiskers (CNWs) to glucose–gelatin films via a Melad cross-linking reaction enhances the film’s thermal stability, ultraviolet absorption, and tensile strength [79]. Chitosan hydrochloride (CHC) and carboxymethyl chitosan (CMC) were used as raw materials to prepare anthocyanins (ACN) nanocomplexes, which were added to gelatin to prepare composite gel films, which showed a reduction in moisture content (MC) from 33.04 to 27.90%, an increase in the melting temperature from 194.03 to 211.30 °C, and an enhancement in the thermal stability and durability of the films [80]. ZnO and clove essential oil (CEO) were doped into bovine skin gelatin (BSG), and semi-transparent gel films were successfully prepared in Petri dishes containing 100 mM CaCl_2_ solution using 3D printing [81].

## 3. Prospects and Challenges

The purpose of food packaging is to provide a barrier for food products from external environmental contamination. There is a need to ensure both the quality and safety of food and to maximize its shelf life. Protein-based active packaging, as a new packaging technology, shows great potential in the food industry. It utilizes the bioactivity and degradability of proteins to achieve multiple goals such as food protection, extended shelf life, and environmental protection. With the increasing global concern for environmental protection and sustainable development, protein-based active packaging is expected to become mainstream in the future. This packaging material can be degraded in the natural environment, reducing environmental pollution and aligning with the trend of green and low-carbon development. Meanwhile, as consumer demand for food quality and personalization increases, protein-based active packaging is expected to be personalized and customized. For example, packaging materials with specific functions are tailored to food types and the tastes and nutritional needs of consumers. Protein-based active packaging technology will become deeply integrated with biotechnology, nanotechnology [82], information technology, and other fields, through the addition of specific enzymes or antibodies, to provide packaging materials with functions such as antibacterial and antioxidant properties and freshness preservation [34]. Nanotechnology or intelligent sensors can be used to achieve real-time monitoring and feedback on the state of the food in the package, meaning that active packaging will take into account functionality and intelligence and thus promote innovative development in the new food packaging industry to meet the needs of more areas.

Currently, the relatively high cost of active protein-based gel packaging and the inadequacy of relevant standards and regulatory systems have limited its wide application in the marketplace. In the future, it will become necessary to improve market competitiveness by making technical improvements and scaling up production costs, as well as to establish a sound standard and regulatory system to ensure product quality and safety. As protein-based gel packaging technology is new and differs significantly to most people’s impression of what plastic film is, consumer awareness and acceptance are limited and thus need improvement by means of the popularization of science and marketing. Subsequently, ensuring the stability and safety of gel packaging materials during storage, transportation, and use to avoid adverse effects on food is also an issue to be considered in the future.

## 4. Conclusions

This paper reviews several plant and animal proteins and some of their applications in food packaging, where proteins can not only be obtained from the plants and animals themselves, but also be recovered from waste by-products. As different proteins have different properties, they possess different film-forming abilities, but the overall approach is similar. Plasticizers, additives, and natural polymers are added to the film production process to improve the mechanical, antimicrobial, and antioxidant capabilities of protein-based films, and the substances added are not harmful to the environment or to the food itself. Protein-based films are also very degradable compared to petroleum-based films, and since the films themselves are protein-based, they are easily biodegradable even when buried in the soil and do not pollute the environment. It meets the needs of modern society for sustainable development. At present, there are still limitations in the types of protein-based films that exist, protein films tailored for specific purposes still need to be further developed, and producing films more efficiently and economically needs further exploration. There is considerable potential for development and in-depth study.

## Data Availability

Not applicable.
